# Ignatia Amara

**Published:** 1891-03-07

**Authors:** 


### THERAPEUTICS.

### IGNATIA AMARA.

A drug closely resembling strychnos nux vomica in its medicinal properties. The seeds from which the drug is prepared are about an inch in length, convex on one side, facetted on the other; are obtained from a medium-sized tree, a native of the Phillipine Isles. The name of the "Bean of St. Ignatius" was applied to it by the Jesuits of that island, who found it highly esteemed by the natives for its medicinal properties. The seeds are very bitter, but have no smell. On analysis they have yielded the same constituents as the strychnos nux vomica seeds, and were found to contain 1·2 of strychnine and a-half per cent. of brucine. Another analysis showed that these alkaloids were united as bases with Igasuric acid, and yield also a volatile principle, extractives, gum, resin, fixed oil, and bassorin. They also contain about ten per cent. of albumoid matter. Obviously, then, ignatia amara must have a *similar* action to nux vomica, and it has, in fact, been proposed as a substitute for that drug. In practice, however, it seems to differ somewhat from nux vomica. Of late it has been our aim to separate the active principles from the more or less indefinite extracts and tinctures, and to use these alkaloids or active principles in their stead. In some cases this is undoubtedly an advantage, but our knowledge of what are really all the active principles in any drug is still too limited to substitute them for the time-honoured tincture, &c. Digitalin certainly cannot be said to represent digitalis, nor does strychnine represent nux vomica. Sometimes the employment of the alkaloids is an advantage, but not always. Our clinical experience with Ignatia amara has certainly not found it to be merely a stronger preparation of nux vomica. Recently our attention has been drawn to the value of strychnine in certain cardiac affections as a heart tonic, especially in combination with digitalis. In those cases in which the heart is atonic, in which the slightest agitation produces a rapid feeble pulse, in which, in fact, there is unwonted nervousness associated with a general deficiency in tone, ignatia proves most valuable given in half-drop to two-drop doses, well diluted, three or four times daily. Our limited experience of the remedy does not enable us to indicate more clearly the conditions in which it is called for, but we think that it is well worth a careful clinical investigation at the hands of the profession.

